# SPPSU/SPES Membranes Reinforced with Electrospun PPSU Mats and Sulfone-Crosslinked: Toward Fluorine-Free Proton Exchange Materials

**DOI:** 10.3390/membranes16040128

**Published:** 2026-03-31

**Authors:** Luca Pasquini, Murli Manohar, Riccardo Narducci, Emanuela Sgreccia, Maria Luisa Di Vona, Philippe Knauth

**Affiliations:** 1 Aix Marseille University, CNRS, MADIREL (UMR 7246) and International Laboratory-Ionomer Materials for Energy, Campus St Jérôme, 13013 Marseille, France; 2 Tor Vergata University of Rome, Department Industrial Engineering and International Laboratory-Ionomer Materials for Energy, 00133 Roma, Italy; riccardo.narducci@uniroma2.it (R.N.);

**Keywords:** sulfonated aromatic polymers, nanofibers, electrospinning, crosslinking, sulfone bridges

## Abstract

Sulfonated aromatic polymers (SAPs) represent promising alternatives to perfluorinated ionomers for proton-exchange membrane fuel cells (PEMFCs), but their high hydrophilicity and limited chemical stability often require structural reinforcement and controlled cross-linking. In this study, composite membranes based on sulfonated poly(phenylsulfone) (SPPSU) and sulfonated poly(ethersulfone) (SPES) were fabricated with and without electrospun PPSU nanofiber mats and subsequently cross-linked through a solvent-induced sulfone-bridge formation at 180 °C. SPPSU/SPES blends (70/30, 50/50, 30/70) displayed good miscibility, while PPSU fibers improved dimensional stability and suppressed excessive swelling. Cross-linking strongly influenced membrane properties: intermediate treatment (20 h) enhanced mechanical strength and solvent resistance with limited loss of IEC, whereas extended treatment (30 h) produced highly stable, low-swelling networks. Despite lower IEC and water uptake, 30 h-treated membranes exhibited higher proton conductivity, attributed to reduced tortuosity and more continuous ionic pathways. Mechanical and hydration analyses identified SPPSU-50, SPPSU-70, and SPPSU-100 as the most balanced compositions. Proton mobility analysis revealed high membrane tortuosity, consistent with dense cross-linked structures reinforced by fibers. Overall, the combined use of SPPSU/SPES blending, PPSU nanofiber reinforcement, and sulfone-bridge cross-linking yields robust, water-insoluble membranes with improved electrochemical performance suitable for PEMFCs and other applications.

## 1. Introduction

Perfluorinated ionomers, such as Nafion and related materials [1,2,3,4,5], present excellent properties, including high ionic conductivity, chemical, thermal, and mechanical stability, making them the current leaders in the field of proton exchange membranes (PEM). However, their environmental impact is a major concern. The manufacturing of perfluorinated ionomers can release toxic byproducts, such as polyfluoroalkyl substances (PFAS), into groundwater and ecosystems. Degradation can also produce PFAS that are toxic and related to hormonal disruption and immune system effects [6]. Being extremely stable, once released, they can accumulate for decades or centuries, becoming so-called “eternal pollutants”. Recycling of perfluorinated ionomers is almost nonexistent.

As an alternative to perfluorinated ionomers, sulfonated aromatic polymers (SAPs) are high-performance materials where sulfonic acid groups (–SO_3_H) are grafted onto an aromatic polymer backbone [7,8]. Common SAPs include sulfonated poly(ether ether ketone) (SPEEK) [9,10], sulfonated polysulfone (SPSU), sulfonated poly(phenylsulfone) (SPPSU) [11], and sulfonated poly(ethersulfone) (SPES) [11]. These materials are particularly important for proton exchange membrane fuel cells (PEMFCs) and biological fuel cells, water purification, nanofiltration membranes, ion exchange applications, and heating, ventilating, and air conditioning (HVAC) systems [12]. Key features of SAPs include high thermal and chemical stability, due to the aromatic backbone, tunable hydrophilicity, and ion conductivity by adjusting the degree of sulfonation (DS). The mechanical strength can be retained even in wet conditions, depending on the base polymer and the sulfonation degree.

However, various challenges must be addressed, including stability vs. conductivity trade-off: a high sulfonation increases conductivity but can lower the mechanical stability and cause excessive swelling [13]. Processing difficulties are also encountered because some highly sulfonated SAPs become water-soluble or hard to cast into membranes.

Various strategies for the improvement of SAPs were discovered over the years, including the formation of composite and blend membranes [14], cross-linking (XL) via diverse pathways [15,16,17], and the introduction of reinforcing fibers, made, for example, by electrospinning [18].

Composite PEMs combine a proton-conductive matrix with inorganic or organic fillers/additives to improve performance. Design strategies include physical mixing of fillers [19,20,21,22,23] and in situ formation of inorganic particles inside the ionomer matrix [24].

Reticulation of SAPs can be based on the addition of cross-linker molecules [15,25,26], such as bisphenol derivatives, or by interchain reactions of particular groups, grafted on the ionomer chain [27]. The latter approach is particularly appealing from an environmental point of view, as it avoids the addition of supplementary small molecules that can be released into the environment. Furthermore, it can be implemented in the membrane formation process [28]. In SAPs, suitably activated sulfonic acid groups can act as electrophiles in an aromatic substitution reaction to form sulfone (–SO_2_–) bridges between adjacent polymer chains. These intermolecular cross-links increase dimensional stability, reduce swelling [29], and improve thermal and chemical resistance, especially under oxidative conditions such as those in fuel cell operation, due to the robust sulfone linkage. Proton conductivity can be maintained or even enhanced, depending on the degree and distribution of sulfonation that governs the continuity of the ionic domains [30]. Cross-linking (XL) of SAPs via sulfone bridges was demonstrated for different ionomers, including SPEEK and SPPSU.

Electrospinning enables the production of nanofibrous mats with high surface area, tunable porosity, and ion-exchange capacity, making them highly suitable for PEMs [18,31,32]. Electrospun mats of non-conductive polymers can be used as reinforcing scaffolds in composite membranes [33]. Electrospun nanofibers of polylactic acid (PLA) have been widely used as biodegradable materials [34]. Successful fiber formation requires careful tuning of solution viscosity and surface tension. The introduction of ionomer-based fibrous scaffolds is also possible; however, high sulfonation levels can be challenging for fiber formation because the conductivity of the solution is increased and the chain entanglement reduced, often leading to bead formation and unstable jets during electrospinning. Blending with non-sulfonated polymers can mitigate these issues by improving entanglement and stabilizing the electrospinning process. Optimization of polymer concentration and electrospinning parameters, such as applied voltage, tip–collector distance, and flow rate, is essential for producing uniform fibers [35]. Common solvent mixtures for SPPSU or SPEEK electrospinning contain dimethyl-acetamide (DMAc), dimethyl-formamide (DMF), or N-methyl-pyrrolidone (NMP), sometimes with added water or alcohol to control viscosity and evaporation behavior. Post-spinning thermal treatments can further enhance the mechanical strength and functional performance of electrospun polymer mats [36], helping to stabilize the fibrous structure and prevent nanofiber loss or dissolution in aqueous environments.

In this work, the combined strategy of blending miscible sulfonated polyphenylsulfone (SPPSU) and sulfonated poly(ether sulfone) (SPES) in varying proportions, incorporating an immiscible electrospun scaffold of unsulfonated PPSU, and adjusting the degree of covalent crosslinking enables the fabrication of low-cost, tailor-made materials whose properties can be readily adapted to diverse operating conditions. The degree of crosslinking was modulated by the time of treatment of the membranes at 180 °C. The water uptake, swelling ratio, ion exchange capacity (IEC), mechanical properties, and ionic conductivity of the plain, fiber-reinforced, and crosslinked membranes were compared.

## 2. Experimental

### 2.1. Materials

Poly(phenylsulfone) (PPSU) (MW = 46,173 g/mol) and poly(ethersulfone) (PES) (MW = 67,080 g/mol) were purchased from Solvay. Sulfuric acid, chlorosulfonic acid, dimethyl sulfoxide (DMSO), NMP, acetone, DMAc, DMF, ethanol, and other chemicals were used as received from Merck-Sigma-Aldrich (Milano, Italy).

### 2.2. Synthesis of SPES

SPES was prepared as reported in the literature [37]. PES (10 g, 86 meq) was dissolved in chlorosulfonic acid (99%, 50 mL) and stirred at room temperature (RT) for 20 h to initiate sulfonation. The resulting solution was then poured into 150 mL of concentrated sulfuric acid (96%) and maintained under stirring at RT for an additional 2 h. The reaction mixture was subsequently precipitated in ice-cold water under continuous stirring, yielding a solid product. After standing overnight, the precipitate was washed repeatedly with ice-cold water until the filtrate reached a pH of 5–6, and the obtained sulfonated polymer (SPES) was dried under vacuum at 80 °C for 20 h. The degree of sulfonation and ion exchange capacity were obtained using both ^1^H NMR and acid-base titration. Both methods yielded the same results: DS = 0.82 and IEC = 2.80 meq g^−1^.

### 2.3. Synthesis of SPPSU

The procedure for SPPSU is reported in the literature [11]. PPSU (20 g, 50 meq) was dissolved in concentrated H_2_SO_4_ (96%, 1 L) and stirred at 50 °C for 5 days to complete sulfonation. The resulting solution was then poured into a large excess of ice-cold water under continuous stirring, forming a white precipitate. After standing overnight, the precipitate was filtered and thoroughly washed with cold water until the filtrate reached neutral pH. The obtained sulfonated polymer (SPPSU) was finally dried under vacuum at room temperature for 5 h. DS and IEC evaluated using both ^1^H NMR and titration were DS = 1.18 and IEC = 2.37 meq g^−1^. Fenton tests of crosslinked SPPSU membranes at 25 °C for 5 h and 75 h show a high stability with a weight loss of 8% and 14%, respectively.

### 2.4. Fabrication of SPPSU-SPES Membranes Reinforced with PPSU Nanofibers

**Figure 1:**
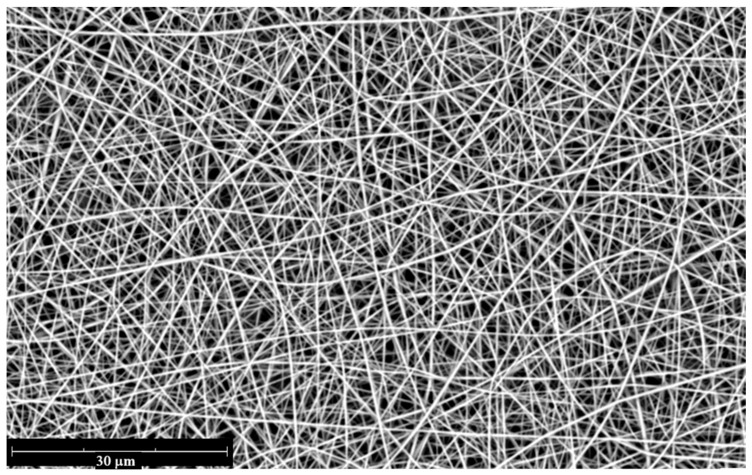
SEM image of the PPSU nanofiber mat.

*Electrospinning of PPSU fibrous mats:* PPSU (23 wt%) was dissolved in an NMP/acetone mixture (75:25 *v*/*v*) to prepare the spinning solution, which was then loaded into a 10 mL syringe fitted with a 0.8 mm diameter needle. The fibers were collected on aluminum foil wrapped around a stainless-steel plate (20 × 20 cm, Linari Engineering SRL, Pisa, Italy). The applied voltage was set to 12 kV, with a tip-to-collector distance of 17 cm. The solution feed rate was maintained at 0.2 mL h^−1^. These parameters, including polymer percentage and mixed solvent ratio, were adapted and optimized in previous works [38,39]. Electrospinning was conducted under controlled environmental conditions of 25 °C and 40% relative humidity. The resulting nanofibrous mats had a typical size of 15 × 15 cm^2^ with an average thickness of 10–15 µm. A typical SEM micrograph (Phenom XL G3, Thermo Fisher Scientific, Waltham, MA, USA) of the electrospun fibers is shown in Figure 1.

*Membrane casting:* SPPSU/SPES solutions (250 mg) were prepared by dissolving the polymers in water (90%) at 40 °C under continuous stirring for 30 min. After complete dissolution, DMSO (10%) was added, and the mixture was stirred for an additional 10–15 min. The casting process was carried out by first spreading a thin film of the polymer casting solution onto a cleaned Petri dish. A layer of porous PPSU nanofibrous mat (≈7 wt%) was then placed on top of the solution, followed by a second spread layer of solution to ensure full embedding of the fibers within the polymer matrix. Composite membranes were prepared with SPPSU/SPES weight ratios of 70/30, 50/50, and 30/70, and electrospun PPSU nanofibers. For comparison, corresponding non-reinforced membranes (“without fibers”) were also cast from the same polymer solutions under identical conditions. The resulting composite films were dried in an oven at 60 °C for 10 h. Thermal cross-linking was subsequently achieved by treating the membranes at 180 °C for 20 h or 30 h, respectively.

### 2.5. Water Uptake and Swelling Ratio

The water uptake *WU* was quantified using the following equation:

(1) $$WU=\frac{W_{w}-W_{D}}{W_{D}}$$

where *W_w_* is the weight of the wet membrane (after 24 h at 25 °C in ultrapure water), and *W_D_* is the weight of the dry membrane (after 24 h at 100 °C).

Moreover, the swelling ratio SR was calculated from Equation (2):

(2) $$\mathrm{SR}\;=\;\frac{L_{s}\;-\;L_{d}}{L_{d}}$$

where *L_s_* is the thickness of the wet sample, and *L_d_* is the thickness of the dry sample, respectively. The area did not change during the immersion in water.

### 2.6. Ion Exchange Capacity

The IEC of the membranes was determined using acid-base titration. The samples in protonated form were dried over P_2_O_5_ for 3 days and weighed; they were then immersed in 20 mL of 2 M NaCl for ion exchange (H^+^ vs. Na^+^). Afterwards, the solutions were titrated with 0.01 M NaOH to reach the equivalence point. The IEC (meq g^−1^) of membranes was calculated from Equation (3):

(3) $$IEC=\frac{C_{sol}\times V_{sol\;}}{W_{D}}$$

where *C_sol_* and *V_sol_* are the concentration and the consumed volume of NaOH.

### 2.7. Conductivity

The in-plane proton conductivity of the membranes was measured using samples of 3 cm × 0.5 cm with a thickness of 40–60 μm. Each sample was mounted in a 4-probe conductivity cell (850 Fuel Cell System, Scribner, Scribner, NE, USA). Measurements were performed by AC impedance spectroscopy (VMP3, Biologic science instruments, Seyssinet-Pariset, France) in the frequency range of 1 Hz–1 MHz with an AC amplitude of 20 mV under 95% relative humidity. The bulk resistance R of the membrane was obtained directly from the intercept with the real axis. The proton conductivity (σ) was calculated from Equation (4):

(4) $$\sigma \;=\;\frac{d}{R\;\times W\times \;L}$$

where σ is the proton conductivity of the membrane (in S cm^−1^), d is the distance between potential-sensing electrodes (0.425 cm), W is the width (0.5 cm), and L is the thickness of the sample.

The through-plane proton conductivity of the membranes was measured using electrochemical impedance spectroscopy (EIS) over the frequency range of 1 Hz–6 MHz with a sinusoidal voltage amplitude of 20 mV, employing a Biologic VSP300 potentiostat. Prior to the measurement, the membranes were immersed in 1 M H_2_SO_4_ and thoroughly washed in water to guarantee full humidification. The samples were then gently wiped to remove excess surface water and mounted in a Swagelok cell equipped with two stainless steel electrodes with a contact area of A = 0.264 cm^2^. The through-plane conductivity (σ) was calculated from the bulk resistance (R), obtained from the impedance spectra, using Equation (5):

(5) $$\sigma \;=\;\frac{L}{R\;\times \;A}$$

L is the membrane thickness.

**Tensile strength.** The mechanical properties of the membranes were determined using a Testometric M250-2.5 CT tensile testing machine (Rochdale, UK). Stress–strain curves were recorded, and Young’s modulus was determined from the slope of the linear region of each curve. The membrane samples were cut into rectangular strips of 5 mm width and ~50 mm length, with an active gauge length of ~25 mm. The thickness of each sample was measured at five different points using a Mitutoyo 293–230 micrometer, and the average value was used for subsequent calculations. The samples were mounted on a tensile testing machine using mechanical clamps, and the measurements were performed at a crosshead speed of 5 mm·min^−1^. All experiments were carried out under controlled conditions of (25 ± 2) °C and (50 ± 10)% relative humidity (RH).

## 3. Results

**Figure 2:**
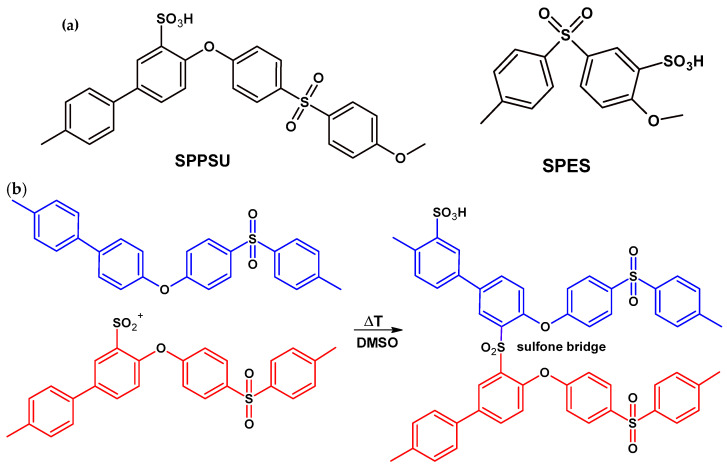
(**a**) Repeat unit of SPPSU and SPES with DS = 1. (**b**) Schematic of SPPSU cross-linking mechanism.

Figure 2a shows the repeat units of SPPSU and SPES.

Membranes based on SPPSU and SPES were prepared with several concentrations, with and without PPSU nanofiber reinforcement. Reinforced membranes were obtained by embedding PPSU nanofiber mats into SPPSU/SPES blends with weight ratios of 70/30, 50/50, and 30/70, while non-reinforced membranes with the same compositions were fabricated for comparison. The homogeneous casting solutions and uniform film morphology confirmed the good miscibility of the two sulfonated polymers.

Crosslinking between sulfone groups occurs when membranes cast in DMSO are subjected to thermal treatment, as previously demonstrated [40]. Figure 2b shows the simplified pathways for the formation of sulfone linkages. The bridging process proceeds through an electrophilic aromatic substitution (S_E_Ar) mechanism, specifically a Friedel–Crafts type acylation involving the formation of a Wheland intermediate [23,24]. The process is mediated by the presence of polar aprotic non-basic solvents like DMSO. Structural investigations systematically exploring the reaction conditions for solvent-induced S_E_Ar have confirmed that XL structures can only be formed when the polymer is in its protonated form (H^+^) and subjected to a thermal treatment above 160 °C [23,24]. DMSO is a polar aprotic solvent with a strong tendency to accept protons and a high dielectric constant (ε = 46.7), originating from the highly polar sulfur–oxygen bond. In addition, DMSO exhibits both nucleophilic and electrophilic character and remains thermally stable under the applied treatment conditions. Previous studies have shown that DMSO interacts strongly with sulfonic acid groups in benzenesulfonic acid, with up to 2.5–3 DMSO molecules coordinating to a single –SO_3_H group; this behavior has also been confirmed by our group for SPEEK [40].

The interaction between –SO_3_H groups and DMSO arises from strong hydrogen-bonding and dipole–dipole interactions. This complex can subsequently evolve into an ion-pair intermediate and ultimately generate the electrophilic sulfonium species that initiates the sulfone-bridge cross-linking reaction. The solvent-induced sulfone crosslinking mechanism has been extensively investigated and experimentally validated in our previous work [40], using complementary techniques including FTIR, thermal analysis, and controlled model reactions.

Before XL, all plain membranes appeared completely transparent, whereas the reinforced membranes were less transparent due to the embedded PPSU nanofiber mat. All membranes could be easily detached from the Petri dish after drying, except for the pristine SPES-100, which adhered strongly to the substrate due to its high hydrophilicity. Initial stability tests after cross-linking at 180 °C for 20 and 30 h were performed by soaking the membranes in water and 1 M H_2_SO_4_, followed by assessment of weight loss and surface appearance. The SPES-100 and SPPSU-30/SPES-70 reinforced membranes exhibited partial dissolution in water and sulfuric acid, indicating poor chemical and structural stability due to their high SPES content. The fact that SPES does not crosslink in these conditions due to the deactivated aromatic rings is well-documented in the literature [41].

The same soaking test on the plain membranes confirmed higher water uptake and swelling compared to their reinforced counterparts, likely due to the absence of cross-linking. Among the reinforced membranes, SPPSU-50, SPPSU-70, and SPPSU-100 represent the most balanced compositions, providing an effective compromise between proton conductivity and structural stability, and are therefore suitable candidates for further electrochemical evaluation.

**Figure 3:**
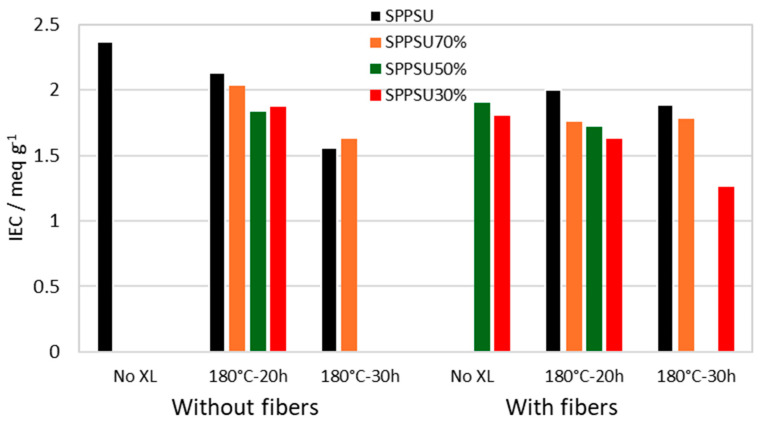
Ion exchange capacity of various membranes.

Figure 3 shows the ion exchange capacity (IEC) of various membranes.

The membranes containing PPSU fibers have a lower IEC due to the presence of the non-sulfonated polymer. The IEC also decreases with increasing crosslinking time, consistent with the reticulation mechanism by SO_2_ bridges involving sulfonic acid groups.

**Figure 4:**
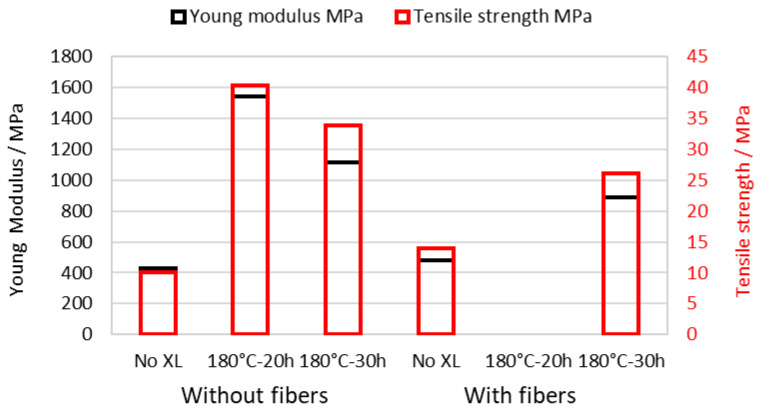
Stiffness and strength of SPPSU membranes with the addition of PPSU fibers and XL by SO_2_ bridges.

As shown in the case of SPPSU in Figure 4, the membrane stiffness (given by the Young modulus) and strength can be modified by the addition of PPSU fibers and by crosslinking.

One can note that fibers reduce the Young modulus and tensile strength for cross-linked membranes, indicating that the fibers are only weakly bound; however, they enhance the mechanical properties of non-XL samples, confirming an alternative pathway for membrane stabilization. The cross-linking treatment for 20 h shows the maximum increase in Young modulus and tensile strength; longer times reduce the stiffness and strength, probably due to a decrease in chain entanglements in the membranes.

**Figure 5:**
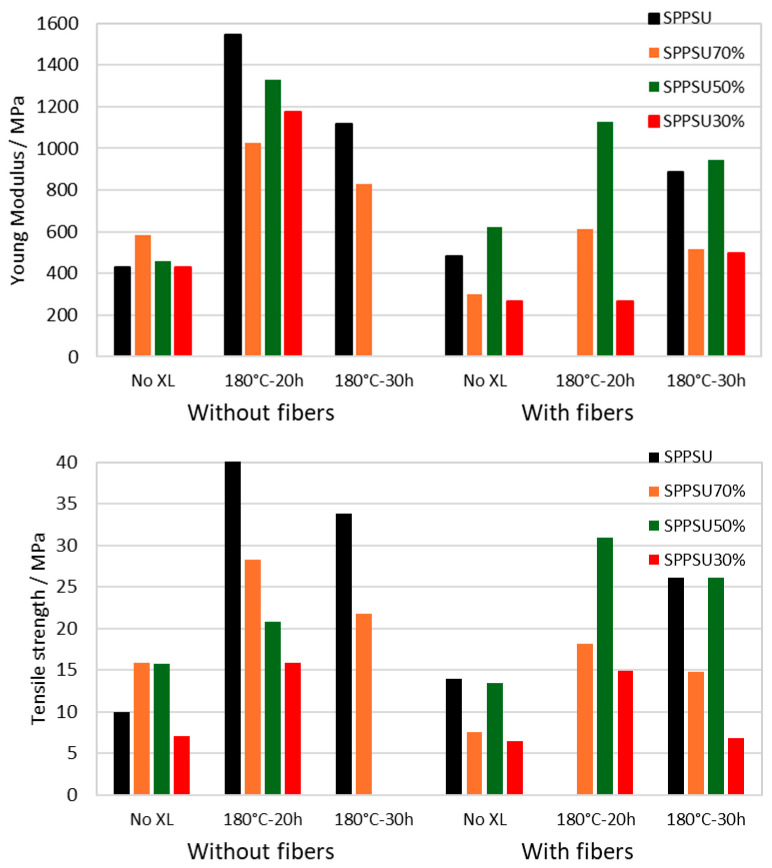
Young Modulus and tensile strength of SPPSU-SPES membranes with/without PPSU fibers and crosslinking treatment.

Similar observations can be made for the blends as shown in Figure 5.

The maximum improvement is observed consistently for 20 h crosslinking treatment, whereas fibers decrease the stiffness and strength of crosslinked membranes. The data for elongation at break are more scattered and show no distinct trend, but pure SPPSU shows a slightly higher elongation at break than membranes containing SPES.

For non-crosslinked materials, the presence of PPSU fibers increases the mechanical properties, indicating good compatibility between the matrix and the reinforcement, probably due to favorable physical interactions between the sulfonated matrix and the aromatic PPSU fibers, such as dipolar and π–π interactions, which enhance stress transfer and restrict chain mobility [42]. The presence of the fibrous scaffold effectively reduces the free volume and limits segmental motion, resulting in increased stiffness and strength.

In contrast, for crosslinked membranes, the addition of non-sulfonated PPSU fibers does not lead to further mechanical reinforcement. In this case, the rigid crosslinked network already limits chain mobility. Moreover, the crosslinking process may induce local heterogeneities at the fiber–matrix interface, leading to reduced interfacial adhesion or partial phase separation between the crosslinked material and the reinforcement [14]. This effect can result in the formation of interfacial voids or weakly bonded regions, which negatively affect stress transfer and ultimately reduce the mechanical performance of the composite.

**Figure 6:**
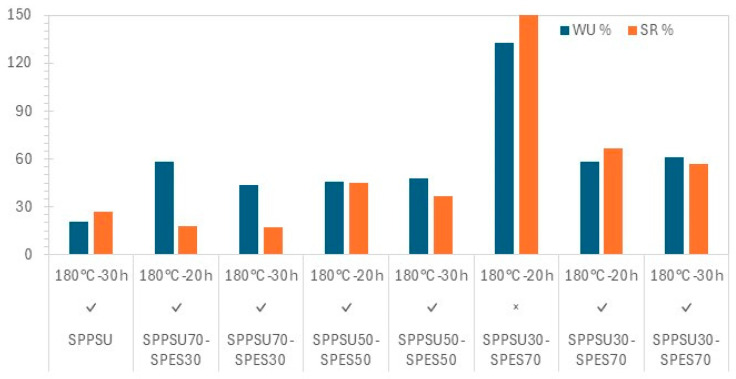
Water uptake and swelling ratio at 25 °C for various crosslinked SPPSU/SPES membranes (×: no fibers, √: with fibers).

Figure 6 shows the water uptake (WU) and swelling ratio (SR). In accordance with the lower mechanical properties, WU is higher for SPPSU-30, which contains very hydrophilic SPES. For comparison, one membrane containing no fibers shows a much higher WU and SR, indicating the relevance of the fiber introduction for control of hydration.

**Figure 7:**
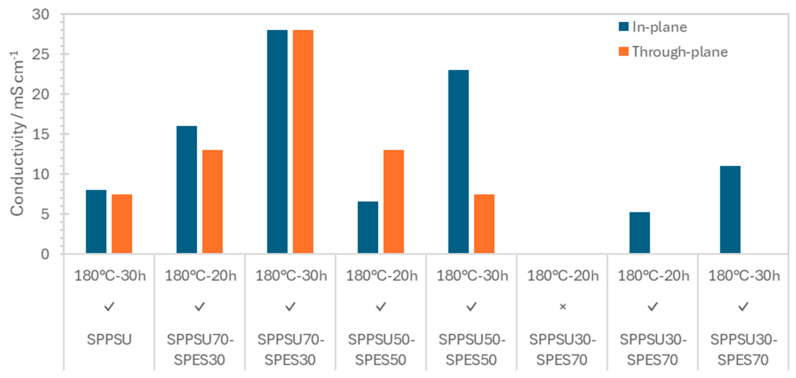
In-plane and through-plane proton conductivity at 25 °C of various membranes (×: no fibers, √: with fibers).

Figure 7 shows the proton conductivity results. The conductivity of the only membrane without fibers cannot be determined, because the large swelling causes loss of contact. There is no apparent difference between in-plane and through-plane data, indicating a homogeneous distribution of ionic domains and demonstrating that the ion-conducting network remains well interconnected and macroscopically isotropic despite the presence of fibers.

Although membranes crosslinked at 180 °C for 30 h exhibit lower IEC and water uptake, they show higher proton conductivity, probably due to the formation of well-defined, continuous ion-conducting channels and reduced swelling. The dense, crosslinked network improves dimensional stability and maintains the connectivity of hydrophilic domains, allowing protons to move more efficiently. Thus, even with fewer sulfonic acid groups, the optimized morphology and lower tortuosity can compensate for the lower IEC, resulting in enhanced overall conductivity. The proton conductivity passes through a maximum for SPPSU-70, reaching nearly 30 mS/cm.

**Figure 8:**
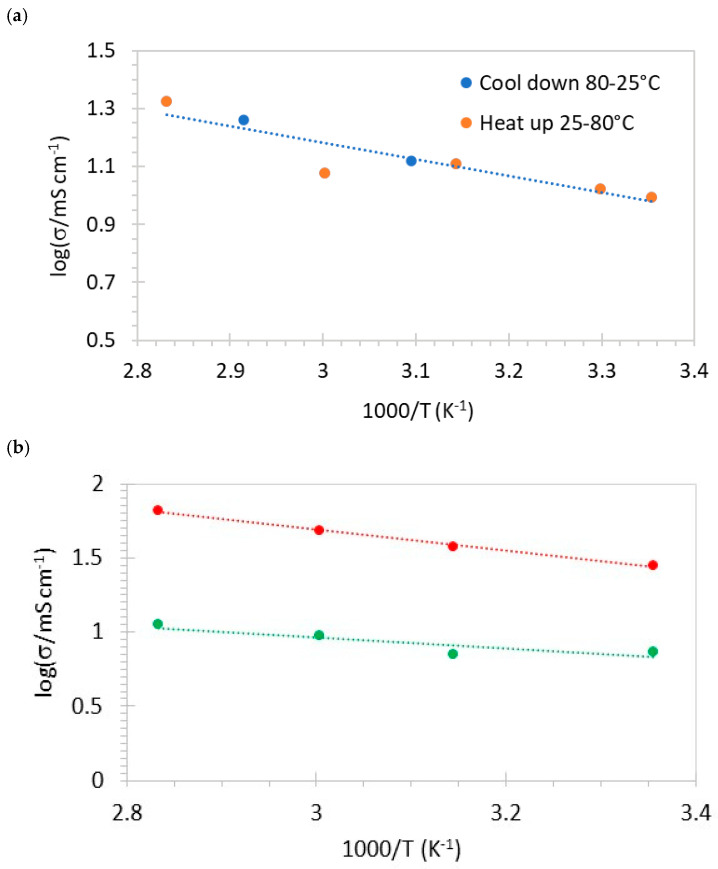
Arrhenius plot of proton conductivity of membranes containing fibers and crosslinked during 30 h. (**a**) SPPSU-100, and (**b**) SPPSU-70 (red) and SPPSU-50 (green).

A typical Arrhenius plot of the through-plane conductivity of SPPSU containing fibers and crosslinked for 30 h is shown in Figure 8a. One can notice that the conductivity measured during heating and cooling cycles is consistent, and no hysteresis is observed. The activation energy for SPPSU with fibers is 11 kJ/mol. The activation energies for the blends (Figure 8b) are quite similar, with an average value for all membranes of (11 ± 5) kJ/mol.

## 4. Discussion

Both SPPSU and SPES can achieve high IEC due to the short repeat unit of SPES and the ability to introduce multiple sulfonic acid groups per repeat unit in SPPSU. However, their high hydrophilicity limits their direct use as membranes for PEMFCs and in aqueous media. In particular, pristine SPES does not undergo thermal cross-linking, likely due to the low reactivity of its sulfonated aromatic rings toward electrophilic aromatic substitution. SPPSU, in contrast, is more hydrophobic and rigid, which can reduce the proton conductivity at relatively low temperatures.

Combining these two polymers, flexible, hydrophilic SPES with mechanically robust SPPSU, enables the formation of better-defined hydrophilic and hydrophobic domains, improving both conductivity and stability. This effect is further enhanced by the introduction of PPSU fibers. Through these interactions, a homogeneous, water-insoluble, and structurally stable membrane is obtained.

Crosslinking time is a key parameter in tuning membrane performance. As crosslinking consumes sulfonic groups, it leads to a decrease in IEC. Therefore, a balance must be achieved: specifically, an intermediate crosslinking time (≈20 h) leads to a moderate decrease in IEC. This condition also improves dimensional stability and solvent resistance, while enhancing mechanical strength without causing loss of entanglements. A longer time (≈30 h) results in extensive crosslinking, producing a denser network. This treatment achieves excellent dimensional stability and solvent resistance and further reduces swelling. However, tensile strength is reduced due to restricted chain mobility. The proton conductivity increases even though the IEC decreases due to the reduction in membrane tortuosity.

The proton mobility in SPPSU-SPES can be determined from the experimental IEC, WU, and proton conductivity. One can first calculate the proton concentration by assuming that the total water uptake is located in hydrated channels where the dissociated protons are present. Any deviation from this assumption, due, for example, to the presence of water outside the hydrated proton-conducting domains or an incomplete dissociation of sulfonic acid groups, will be evidenced by this approach, as shown in previous work [43,44].

The proton concentration c(H^+^) (mmol/cm^3^ = mol/L) can be calculated from the IEC (meq/g) and the WU (%) using the following equation:

(6) $$c\left(H^{+}\right)=100\times \frac{IEC}{WU}$$

For the calculation, the density of the acidic solution is taken as 1 g/cm^3^.

The proton mobility u(H^+^) (cm^2^∙s^−1^∙V^−1^) is defined by the proton conductivity σ(H^+^) (mS.cm^−1^) and the proton concentration according to the equation:

(7) $$u\left(H^{+}\right)=\frac{\sigma \left(H^{+}\right)}{c\left(H^{+}\right)\times F}$$

F is Faraday’s constant (96,485 C∙mol^−1^).

**Figure 9:**
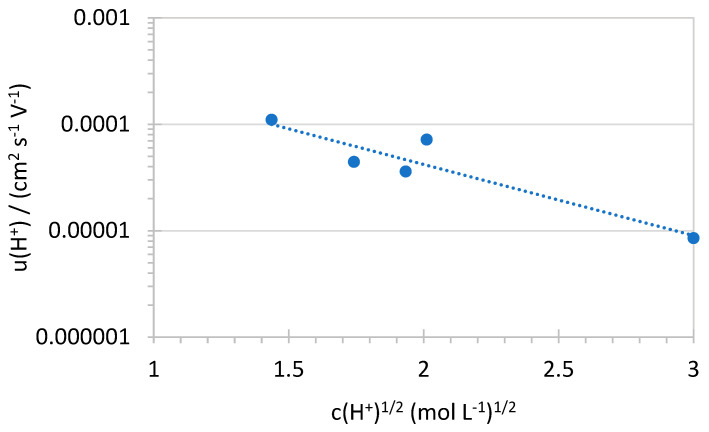
Proton mobility dependence for SPPSU/SPES membranes.

The obtained proton mobility for various membranes is plotted against the square root of the proton concentration in Figure 9. One recognizes a strong concentration dependence, similar to that observed for weak electrolytes in aqueous solution [45].

The extrapolated proton mobility *u* at infinite dilution (Figure 9: 9 × 10^−4^ cm^2^ s^−1^ V^−1^) in SPPSU/SPES blend membranes can be compared with the proton mobility at infinite dilution in water *u*° (3.6 × 10^−3^ cm^2^ s^−1^ V^−1^). The renormalization Equation (8) relates the proton mobility to the membrane porosity π and tortuosity τ:

(8) $$\frac{u}{u{}^{\circ}}=\frac{\pi }{\tau }=0.25$$

The Bruggeman relation between porosity and tortuosity is as follows [46]:

(9) $$\tau =\pi^{-\left(\alpha -1\right)\;}$$

where α is the universal percolation exponent. Assuming the value for the 3-dimensional percolation, α ≈ 2, one can calculate the average membrane tortuosity: τ = 2.0. This value is clearly higher than that determined for SPEEK (τ = 1.28 [47]), showing a high tortuosity of cross-linked SAP membranes containing a PPSU reinforcement mat, although XL is lowering the tortuosity. Strategies to further reduce membrane tortuosity include optimizing the degree of crosslinking to preserve continuous hydrophilic pathways, controlling nanofiber content and orientation to guide ion transport, and promoting controlled phase separation to shorten proton-transport pathways while maintaining mechanical stability.

## 5. Conclusions

In this work, novel blend membranes composed of highly sulfonated SPPSU and SPES were realized. By varying the polymer ratios (70/30, 50/50, 30/70) and introducing electrospun PPSU fiber reinforcement combined with thermal cross-linking (180 °C for 20 or 30 h), we systematically evaluated strategies to enhance the mechanical and hydrolytic stability of the membranes. Unlike previous studies focusing on either blending, fiber reinforcement, or cross-linking individually, this work integrates all three strategies in a single membrane architecture, enabling simultaneous control of dimensional stability, mechanical integrity, and ionic conductivity.

The decrease in IEC with increasing XL time confirms the formation of sulfone bridges through reactions involving sulfonic acid groups. Mechanical stiffness and strength reach an optimum after 20 h of crosslinking, while in the absence of crosslinking, the electrospun PPSU fibers alone already provide significant mechanical reinforcement. Hydrolytic stability is likewise improved by fiber incorporation. An improvement of the interface bonding in XL membranes, perhaps by functionalizing the PPSU fibers, might further reinforce rather than weaken the structure. Performance peaks at 20 h for strength but at 30 h for conductivity, suggesting a more efficient heating protocol to obtain both high strength and high conductivity at once. This point will be further investigated in future studies.

Proton conductivity is highest for membranes with an SPPSU-70 composition, and the activation energies remain low (10–15 kJ/mol). XL reduces membrane tortuosity, although values remain higher than those of pure SPEEK. Future work should focus on further decreasing tortuosity to achieve proton conductivities compatible with the stringent requirements of electrochemical energy and water treatment technologies.

## Data Availability

The raw data supporting the conclusions of this article will be made available by the authors on request.
